# Bird and mammal diversity in agricultural landscapes and adjacent natural vegetation of the Brazilian Atlantic Forest: a systematic map

**DOI:** 10.1186/s13750-026-00390-z

**Published:** 2026-07-03

**Authors:** Fábio Casallanovo, Gustavo Souza Santos, Ana Paola Cione

**Affiliations:** Syngenta Proteção de Cultivos Ltda, São Paulo, Brazil

**Keywords:** Avifauna, Mastofauna, Bats, Brazilian Atlantic Forest, Agricultural ecosystems

## Abstract

**Background:**

Tropical areas, particularly Brazil, are home to a significant portion of the world’s biodiversity, with the Brazilian Atlantic Forest (BAF) being a crucial biome recognized as a global biodiversity hotspot. However, the BAF has been subject to severe degradation due to anthropogenic activities, resulting in extensive habitat loss and fragmentation. These changes have prompted numerous studies on their effects on biodiversity, focusing on forest fragments and protected areas. Despite this research, there is a notable lack of meta-data on biodiversity in human-modified landscapes within the BAF- where only 31% of the land remains as native forest. Most of the biome has been transformed by human activities, primarily for pasture and agriculture. By recognizing these knowledge gaps, researchers have begun investigating biodiversity in anthropogenically altered landscapes, to better understand species’ responses to human-induced changes and inform conservation policies. A comprehensive literature search was conducted to answer the following primary question: “*What is the diversity of birds and mammal species in agricultural fields and their adjacent areas of natural vegetation*?” We aimed to address this question by creating a systematic map summarizing current knowledge about avian and mammalian species’ presence in the BAF’s agricultural matrix. This research also aimed (i) to identify common species in agricultural landscapes, (ii) to compare species richness in-crop (inside the agricultural field) and off-crop (non-cultivated areas outside the agricultural field), (iii) to determine crops with high species visitation, and (iv) to highlight areas for future research.

**Methods:**

We conducted a systematic literature map on bird and mammal biodiversity in agricultural landscapes within the BAF and adjacent natural vegetation areas. The search covered Web of Knowledge, Scopus, the National Library of Medicine, and the “*Biblioteca Digital Brasileira*” (Brazilian Digital Library) database for grey literature (dissertations and theses). Citation records were surveyed based on predefined criteria through title/abstract screening and full-text analysis. Eligible publications were used for meta-data coding and systematic map construction.

**Review findings:**

Our systematic map identified 207 eligible publications included in the final evidence base. Research effort was distributed across taxonomic groups, with 78 studies focusing on birds, 126 on non-flying mammals (NFM), and 22 on bats. The most frequently studied agricultural systems were pasture and eucalyptus, followed by sugarcane, coffee, cacao, rubber tree, pinus, grape, araucaria, and corn. Extracted meta-data showed that studies reported species occurrences in both agricultural fields and adjacent natural areas. The map revealed significant knowledge clusters and gaps, particularly regarding the distribution of research across different crop types and the inclusion of species with varying conservation statuses.

**Conclusion:**

This systematic map identifies 846 species across 48 crop types in BAF agricultural landscapes, potentially indicating their use by wildlife; however, further quantitative synthesis is necessary to assess the relative importance of these areas. By mapping knowledge clusters and gaps—such as underrepresented regions and specific crop-taxa associations—this work provides a foundation for future systematic reviews and informs the development of more targeted environmental risk assessments and conservation policies.

**Supplementary Information:**

The online version contains supplementary material available at https://doi.org/10.1186/s13750-026-00390-z.

## Background

Tropical areas comprise most of the world’s biodiversity [1], with Brazil, whose territory is predominantly located in the tropics, standing out, accounting for 20% of the world’s species [2]. These species are distributed among 6 different biomes. The Brazilian Atlantic Forest (BAF) is the third largest biome, occupying 13% of the country’s territory, after the Amazon (49.5%) and the *Cerrado* (23.3%), and extends across 17 of the Brazilian 26 states [3].

The BAF comprises a wide range of distinct ecological formations. Its diverse climatic types and landscapes directly contribute to the different structures of the forest [4]. Due to its high species diversity and endemism, this biome is recognized as one of the world’s biodiversity hotspots [5, 6]. Tropical rainforests worldwide face degradation challenges [7], as the pressure to increase food production leads to the transformation of native areas into agricultural land [8]. The BAF biome is close to highly populated areas [9], where anthropogenic activities such as urbanization, agricultural expansion, and livestock production have resulted in the loss of 88% of its native vegetation [10]; these activities can also lead to habitat fragmentation [11] and probably have contributed significantly to a decline in biodiversity [12].

The effects of this extensive habitat loss and fragmentation on biodiversity have been the subject of numerous studies [13–16], where habitat fragmentation resulted in the formation of forest edges, which in turn can promote changes in the ecosystem’s physical, chemical, and biological parameters [13]. These fragments and their impacts have been the subject of further investigation on species occurrence and diversity both locally [14, 15] and in other countries [16, 17]. Wilmer et al. (2022) [16] conducted a meta-analysis on the subject and identified some trends, comparing data from both temperate and tropical countries. Nonetheless, despite the substantial body of research concerning species diversity within forest fragments and protected areas, there is a lack of basic research in human-modified landscapes [18, 19], which include plantations, agricultural fields, tree crops, and pastures. In Brazil, researchers have attempted to address this knowledge gap [20, 21], aiming to understand the effects of agroecosystems on biodiversity and the landscape components that are important in explaining these effects.

Despite these efforts, there is still a knowledge gap for several biomes, including the BAF [22]. Data from Mapbiomas (2023) [10] indicate that in the BAF Biome, as of 2023, only 31% of the land has native forests. In contrast, over 69% have been altered by anthropogenic activities: pasture (29%), agriculture (20%), a mosaic of land uses (18%), forestry (4%), and other uses (5%). In this context, investigating biodiversity within anthropogenically altered landscapes is crucial for enhancing our comprehension of species’ responses to human-induced modifications and informing environmental management policies to conserve biodiversity in these regions [23]. To address this knowledge gap, based on a previously published protocol [24], we conducted a comprehensive literature search (Fig. 1) based on the guidelines of the CEE. This systematic map to collates and describes the evidence base regarding the presence of avian and mammalian species within the agricultural matrix of the BAF, aiming to identify knowledge clusters and gaps to inform future research and conservation monitoring.

**Fig. 1:**
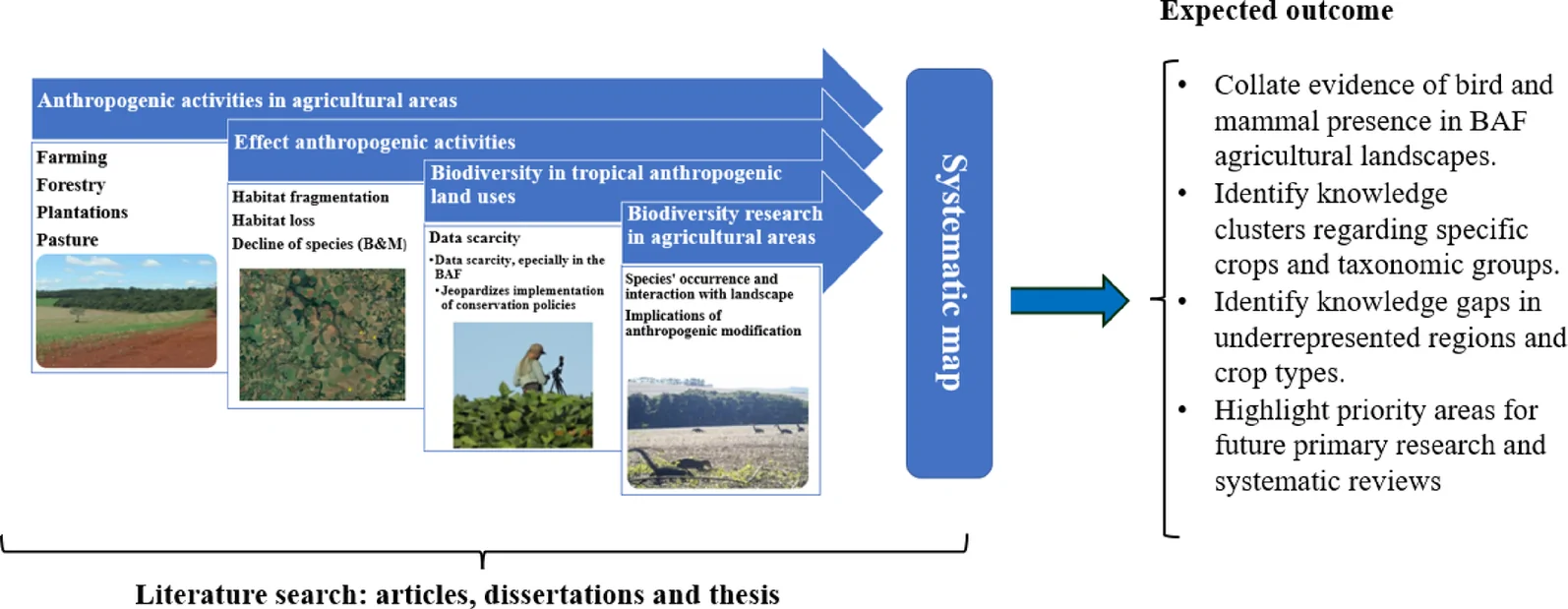
Conceptual model representing the theory of change and the need for a systematic map and the expected outcome. Adapted from original figure published by Casallanovo et al. (2024) [24].

### Objective of the review

This systematic map aims to collate scientific evidence on the presence of birds and mammals in agricultural landscapes and their adjacent areas of natural vegetation, considering the BAF.

#### Primary question

What is the diversity of birds and mammal species in agricultural fields and their adjacent areas of natural vegetation?

In addition to the primary question, we also aimed at addressing the following related sub-questions:

- What are the types of agricultural land use (e.g., annual crops, perennial crops, agroforestry) in the BAF documented in studies of bird and mammal diversity?
- What bird and mammal species are most documented in studies of agricultural areas in the BAF?

#### Definitions of the question components

The question components were addressed using the PICO/PECO model [25], which enables framing the questions of the systematic map according to four elements: Population, Intervention/Exposure, Comparator, and Outcome. Each component is outlined below:

Population (P): birds and mammals (including flying and non-flying mammals and new world marsupials) in the Brazilian Atlantic Forest region.

Exposure (E): agricultural landscapes in the BAF including:

- perennial crops such as coffee, palm trees, cocoa, fruits, etc.
- annual crops such as soybean, corn, rice, wheat, etc.
- agroforestry systems such as cacao-forest, rubber tree-forests, coffee-forests, etc.
- tree plantations such as eucalyptus, pinus, rubber tree, etc.
- pasture and its adjacent areas of natural vegetation (e.g. natural forests, natural fields, riparian areas, etc.).

Comparator (C): adjacent natural areas including natural forests, natural fields, riparian areas.

Outcome (O): species diversity measures including species occurrence patterns, species presence information, the number of publications documenting each species, and the number of publications across agricultural landscapes.

## Methods

The protocol for this systematic map was registered in PROCEED (https://www.proceedevidence.info/protocol/view-result?id=166) as PROCEED-23-00166 and published in *Environmental Evidence* in March 2024 [24]. The systematic map adhered to the Collaboration of Environmental Evidence (CEE) Guidelines and Standards for Evidence Synthesis [25] and conformed to the RepOrting standards for Systematic Evidence Synthesis (ROSES) [26] (Additional file 1).

### Deviations from the protocol

There were five deviations from the published protocol:

(i) Manuscript screening - due to the large number of retrieved records, checking publication availability and extracting PDFs was conducted after title and abstract screening rather than before.
(ii) Data coding strategy - we have the additional components: (a) order and (b) the International Union for Conservation of Nature (IUCN) Red List of Threatened Species [27]. The inclusion of species order allowed us to obtain more detailed taxonomic classification regarding the species that are observed in agricultural fields, while the IUCN data helps map the state of conservation of the species observed in the agricultural fields in the BAF and can contribute to the discussion of protection policies of these species as well as in the discussion of focal species to be used in ERA schemes.
(iii) Definitions of the question components Comparator and Outcome: considering the scarcity of abundance data in the surveyed publications, we focused on species diversity instead of abundance. Therefore, the primary question was adjusted to reflect this deviation.
(iv) Change in the title to more closely align with the primary question/PECO elements.
(v) Secondary questions were modified to better reflect the available data and align with the revised primary question focusing on species diversity rather than abundance.
(vi) The published protocol contained elements typically associated with a systematic review, including critical appraisal of study validity, risk of bias assessment, and narrative and quantitative synthesis components. All such elements were removed in the final manuscript to ensure full alignment with systematic map methodology, therefore focusing on describing and cataloguing the available evidence base rather than appraising or synthesizing study findings.

### Search for articles

The literature search was performed without limiting the year, performing searches up to February 2025. Searches were performed in English and Portuguese. Searches in English were performed in the following databases: Web of Science (WoS), Scopus, and the National Library of Medicine (PubMed). Searches were performed in Portuguese in the *Biblioteca Digital Brasileira de Teses e Dissertações* (BDTD, Brazilian Digital Library of Theses and Dissertations).

#### Search string

The search string used in both languages is indicated below, the search string was developed based on ten benchmark articles, after analyzing the PECO components, and considering the comments from the reviewers of the protocol. Additional details on how the search string was developed can be found in the protocol [24] and search settings on Additional file 2.

Search string in English (WoS, Scopus and PubMed):

(bird* or mammal* or avifaun* or primate* or rodent* or avian or marsupial* or faun* or omnivor* or carnivor* or frugivor*, granivor*or insectivor* or bat* or passerin* or chiropter*) and (agricult* or crop* or farm* or pasture or matrix or patch* or corridor* or “protected area” or plantation* or fragment* or tropic* or ecosystem* or agroforest* or “secondary forest*” or “forest remnant*” or “agricultural field*” or “primary forest*” or arabl* or system* or practice* or management or organic or agroecolog* or “conservation agriculture” or “pest management” or biocontrol or “urban agriculture” or till* or abandonment or set-aside or fallow* or “mixed croplivestock*” or “integrated crop-livestock*” or “diversified crop-livestock*” or “vegetation strip*” or “insect strip*” or “flower strip*” or diversification or rotation or “inter-crop” or covercrop*) and (“atlantic forest” or “brazilian atlantic forest” or riparian or semi-deciduous or “brazilian atlantic rainforest*” or national park* or tree plantation* or perenn* or “perennial crop*” or neotropic*) and not (caatinga or cerrado or amazon or pampa or argentina) and brazil.

Search string in Portuguese (BDTD):

(ave or avifauna or passeriforme or mamífero or masto or roedor or primata or marsupial or fauna or insetivor* or carnívor* or granívor* or frugívor* or onívor* or morcego or quiróptero) and (agricultura or agro* or fazenda or cultivo or plantação or past* or matriz or fragmento or corredor or “àrea protegida” or tropical or agrofloresta* or ecosistema* or “floresta secundária” or “floresta primária” or “floresta remanescente” or “campo agrícola” or arável or sistema or prática or manejo or orgânic* or agroecolog* or “manejo de pragas” or biocontrole or “plantio direto” or pousio or rotação or diversificação or “cobertura vegeta*” or “agricultura urbana” or “faixa de flores” or “faixa de insetos” or “faixa de vegetação” or “cultura de cobertura” or “sistema lavoura-pecuária” or “integração lavoura-pecuária” or “lavoura-pecuária diversificada”) and (“mata atlântica” or “floresta atlântica” or perene or “cultivo perene” or neotropical or semidecídua* or ripária not caatinga not cerrad* not amazônia not pampa not amazon* not argentina).

### Article screening and study eligibility criteria

#### Screening process

Before commencing the screening process, we conducted a pilot screening test between reviewers (FCA and GSS) to check for consistency, as indicated by the study protocol [24]. The procedure was as follows:

- Both reviewers independently screened 200 records, approximately 10% of the total citation records.
- Both reviewers’ eligibility decisions were compared, and consistency was checked by calculating Cohen’s Kappa index (k) [28].

The result of this check yielded a k value of 0.87, which indicates an acceptable agreement [29]; therefore, no further consistency test was necessary. The pilot test is also available as Supplemental Material (Additional file 3).

All literature searches and the retrieved records (articles, theses and dissertations) were combined using Rayyan’s systematic review tool [28], where duplicates were identified using the duplicate feature, and duplicates were removed by direct comparison. After the deduplication process, citation records were then initially screened by title and abstract according to the eligibility criteria. After that, all records were exported to an Excel spreadsheet (see Additional file 4), where a unique manuscript identification number (record ID) was attributed to each record to facilitate tracking. If records were not excluded based on the eligibility criteria, then we proceeded to the next screening phase – full-text availability. Records that were not available were excluded, while those available were screened by reading the full-text and comparing them to the eligibility criteria. The reasons for manuscript exclusion at each stage were recorded in Additional file 4; this file also contains a codebook sheet which contains additional information.

#### Eligibility criteria

In each step, citation records’ eligibility was evaluated according to the following questions, aimed at addressing the PECO components:

1. Does the study include birds or mammals? (PECO component: population)
2. Was the study conducted in the BAF? (PECO component: exposure)
3. Was the study conducted in an agricultural landscape (including its adjacent natural areas)? (PECO component: comparator)
4. Does the study measure diversity? (PECO component: outcome)

Studies were discarded if they received any “no” answers, with reasons for exclusion recorded.

#### Study validity assessment

No formal study validity assessment was performed on the eligible studies, as the systematic map aimed to collate occurrence meta-data. The evidence base consists primarily of observational studies.

#### Data coding strategy

Studies were coded in an Excel file with a codebook as indicated in the protocol [24] (*Additional file 5*) and in the deviations from the protocol, aiming to generate an evidence base to address the PECO questions. The codebook consisted of the following elements: species ID code, species, crop, species occurrence off-crop, taxonomic group, Order, IUCN classification, biological organization (bio_organization), agricultural system, ecosystem, CU area, GPS Info, region, study title, study ID, authors, source title, year, abstract, DOI, and study type. The definition of each coding element can be found in the codebook (Figs. 2 and 3).

The retrieval of metadata such as the number of studies per year, species, crop type, taxonomic Orders and ecosystems (Figs. 4, 5, 6 and 7) and a heatmap of (Fig. 8) was performed using the R Studio software. The script is on Additional file 6.

#### Data mapping

The data and metadata of the eligible publications were coded from the text and supplemental material to build the evidence base. The evidence base was then mapped through descriptive analysis, heatmaps, graphs and tables, which were used to describe elements of the PECO questions, as well as bibliometric elements such as the publication type and publication year.

### Review findings

#### Review descriptive statistics

The number of citations records (articles, dissertations, and theses) at each stage of the systematic map process is shown in the ROSES flowchart [29] (Fig. 2). Article searches on WoS, Scopus, PubMed, and Google Scholar retrieved a total of 15,593 records. Most records were retrieved from Scopus (*n* = 6,705), while searches in WoS (*n* = 4,346) and PubMed (*n* = 4,115) yielded similar numbers of records. Google Scholar searches, performed at the beginning of the systematic mapping process to retrieve benchmark articles for defining the search string used in WoS, Scopus, and PubMed, yielded 787 records. Regarding grey literature, the search on BDTD retrieved 4,981 dissertations and theses. All records were consolidated in the Rayyan database, resulting in 19,282 citation records.

**Fig. 2:**
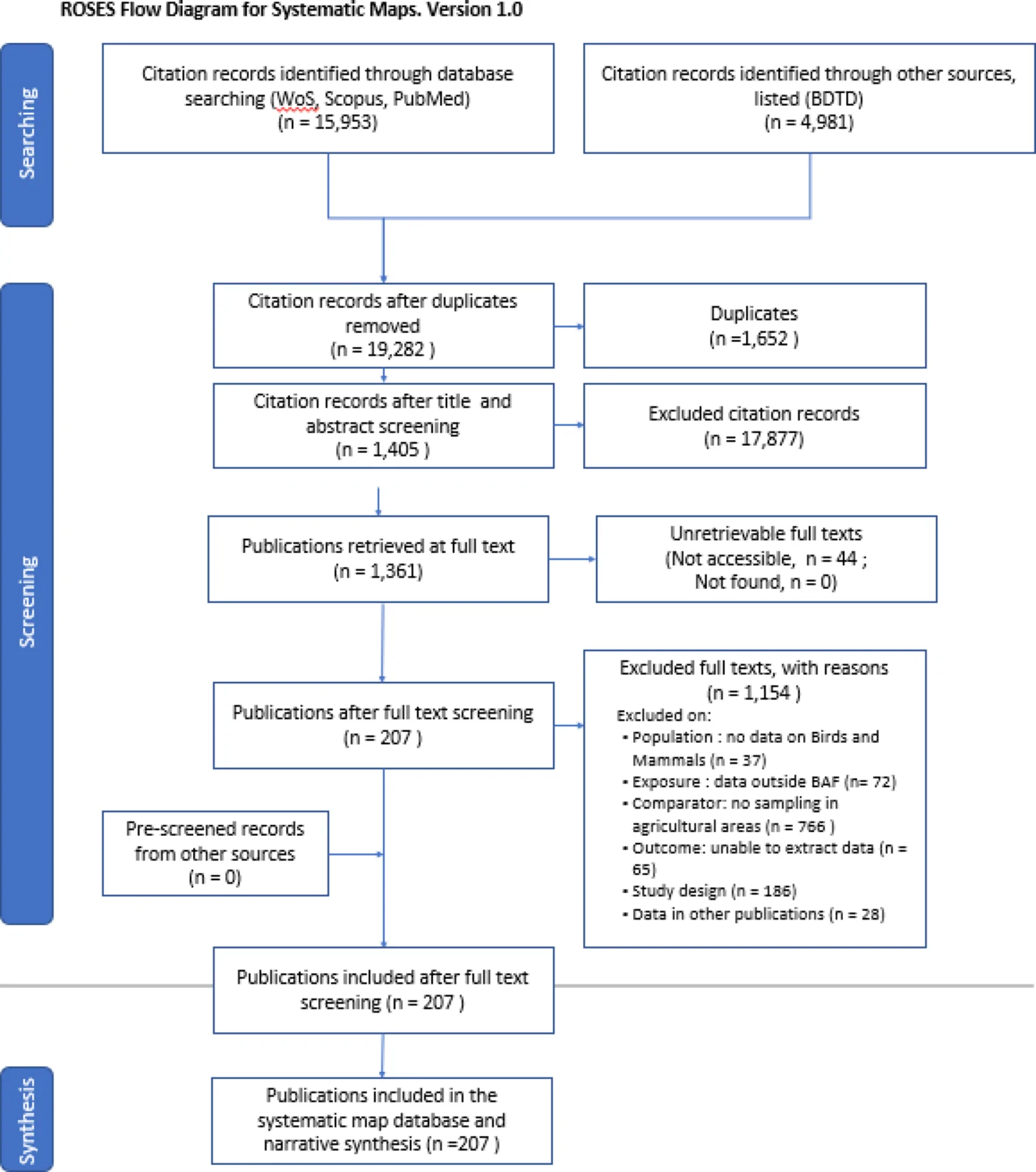
ROSES Flow Diagram summarizing the steps of the Systematic Mapping of the literature (Articles, Dissertations and Thesis) regarding the presence of Birds and Mammals in Agricultural Landscape in the BAF. Version 1.0. Flow chart obtained from Haddaway et al. (2017) [29]

After retrieving all records from the selected databases and consolidating them in the Rayyan software tool [30], we used the software’s deduplication feature to remove duplicate records. As indicated in Fig. 2, a total of 1,652 duplicates were identified and excluded. After duplicate removal, we proceeded with the abstract and title selection, where citation records were evaluated according to the eligibility criteria (*see questions 1 to 4 in Methods*). This process resulted in the exclusion of 17,877 records, while 1,405 records were selected for the next phase: document availability (see Fig. 2). We were unable to retrieve 44 records, leaving 1,371 records for analysis. The remaining documents were again evaluated against the eligibility criteria. If the answer to all the questions was affirmative, the citation record was deemed eligible and was selected for meta-data coding; otherwise, records were excluded, and the reasons for exclusion were recorded (Additional file 4*)*. At this stage, 207 publications were selected for meta-data coding (Fig. 2).

### Characteristics of the evidence base

#### Publication type

Regarding the 207 publications from which the meta-data were collected, 39 (18.8%) correspond to theses and dissertations, while 168 (81.2%) correspond to scholarly peer-reviewed articles. All thesis and dissertations are in Portuguese, while almost all articles (167) are in English.

#### Geographical distribution of studies

As part of the data coding strategy (*see Additional file 5*), we obtained GPS coordinates for eligible study when available. Out of 207 eligible studies, GPS coordinates were not available for 36 publications(17.4%), and the remaining records were then used to map the sampling sites and their distribution across Brazil and the BAF (Fig. 3). Most of the eligible studies are within the boundaries of the BAF, while the sampling points outside the major green area in Fig. 3 correspond to ecotones of Atlantic Forest.

**Fig. 3:**
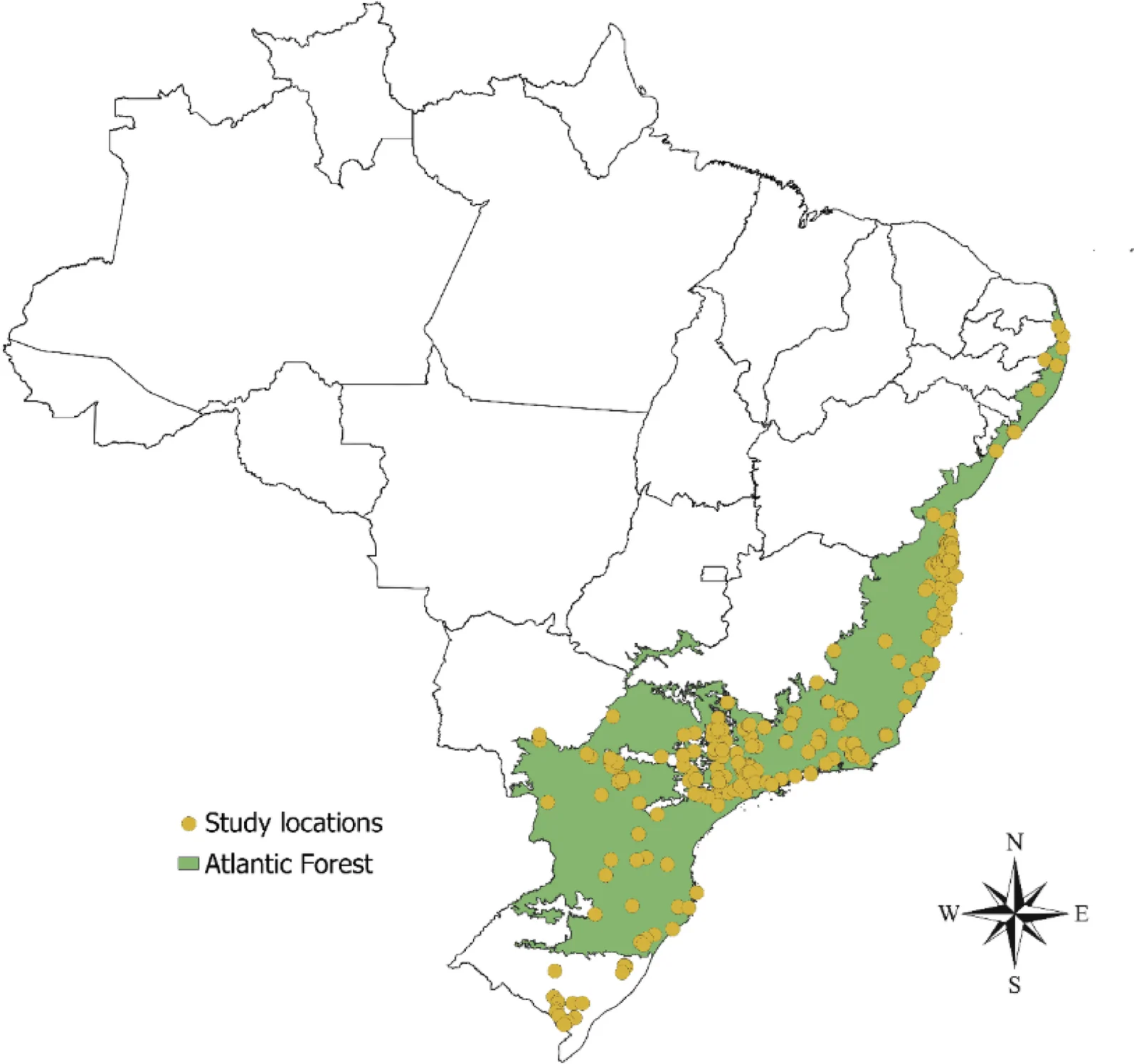
Location of the eligible studies from the eligible publications. In green, areas of the BAF biome. Map constructed using ArcGIS. GPS coordinates are available on Additional file 5

Table 1 shows the distribution of publications across Brazilian states. Most of the eligible studies were in the Southwest, especially in the State of São Paulo. As of 2023 [31], the Southeast of Brazil comprises 39.9% of the area occupied by the BAF in Brazil, while the State of São Paulo contains 14.5% of BAF (*Table **S1*,* Additional file 6*). In the Northeast, most of the studies are in the State of Bahia while in the South of Brazil, the evidence base is fairly distributed across all states. The Northeast contains 21% of the area occupied by the BAF, where the state of Bahia alone accounts for 12.2% of the BAF. There was also one eligible study in the state of Mato Grosso do Sul in the Brazilian Midwest, which represents 4.34 and 4.54%, respectively, of the area occupied by the BAF.

**Table 1:** Geographic distribution of research effort (number of eligible studies) across Brazilian states and regions in the Brazilian Atlantic Forest (BAF), categorized by taxonomic group

Brazilian region/State	Total studies	Studies on birds	Studies on NFM	Studies on bats
Northeast*	59	8	44	9
Alagoas	1	0	1	0
Bahia	49	8	37	5
Paraíba	4	0	3	1
Pernambuco	2	0	2	1
Sergipe	3	0	1	2
Southeast*	***124***	***53***	***67***	***10***
Espírito Santo	7	1	5	1
Minas Gerais	24	12	12	2
Rio de Janeiro	17	3	12	2
São Paulo	76	37	38	5
South*	***32***	***17***	***14***	***3***
Paraná	11	4	6	2
Rio Grande do Sul	10	5	5	0
Santa Catarina	11	8	3	1
*Midwest*	***1***	***0***	***1***	***0***
Mato Grosso do Sul	1	0	1	0
Total (Brazil)*	216*	***78***	***126***	***22***

The total number of unique eligible studies is 207. The sum of studies across states and taxonomic groups exceeds this total because some studies were conducted in multiple states or included more than one taxonomic group. The sum of studies across taxonomic groups exceeds the number of total studies because some studies also included more than one taxonomical group

#### Publication year

The publications from the evidence database were published from 1990 to February 2025 (Fig. 4), increasing from 2008 to 2015 when they reached the peak. After that there was some fluctuation in the number of publications in the following years and a clear decrease from 2022 onwards, coinciding with the years of the COVID pandemic, which might have restricted experimental work.

**Fig. 4:**
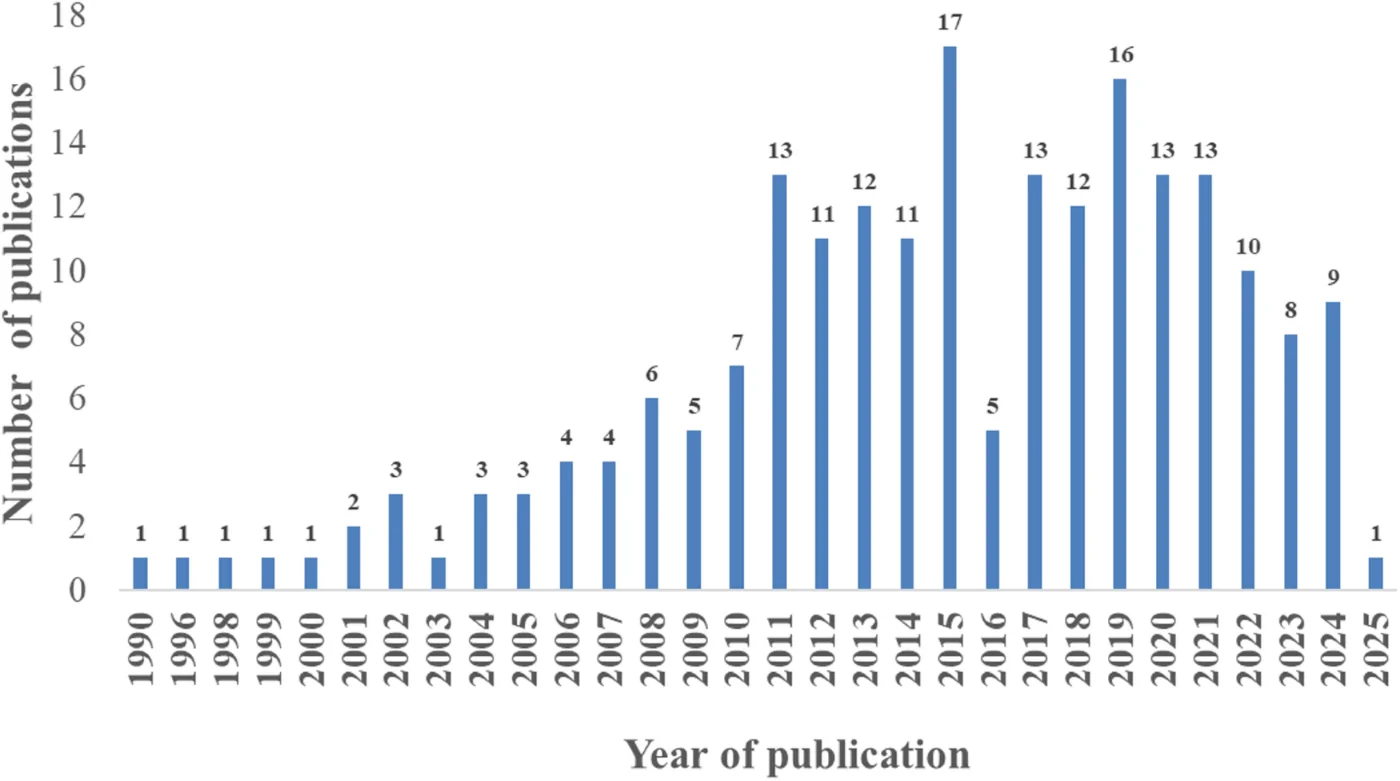
Number of publications per year from the evidence database

### Mapping the quantity of studies relevant to the question

#### Publications according to species and crops

The most studied species were grouped into clusters (Fig. 5), where cluster 1 includes species with more than 20 different publications, cluster 2 includes species between 15 and 20 publications, and cluster 3 which includes species with 10–14 publications (Additional file 7). Cluster 1 shows that only 27 species had more than 20 individual publications, while cluster 2 and 3 gathered 57 and 60 species, respectively. 143 species had between five to nine publications, while the remaining meta-data (559 species) had fewer than five publications each (not plotted in Fig. 5 due to the high amount of meta-data). As for documented crops in the evidence base (Fig. 6), pasture, eucalyptus, cacao, sugarcane and pinus were the five most studied crops. Together, both datasets indicate that experimental research is concentrated in a relatively small number of species and crops.

**Fig. 5:**
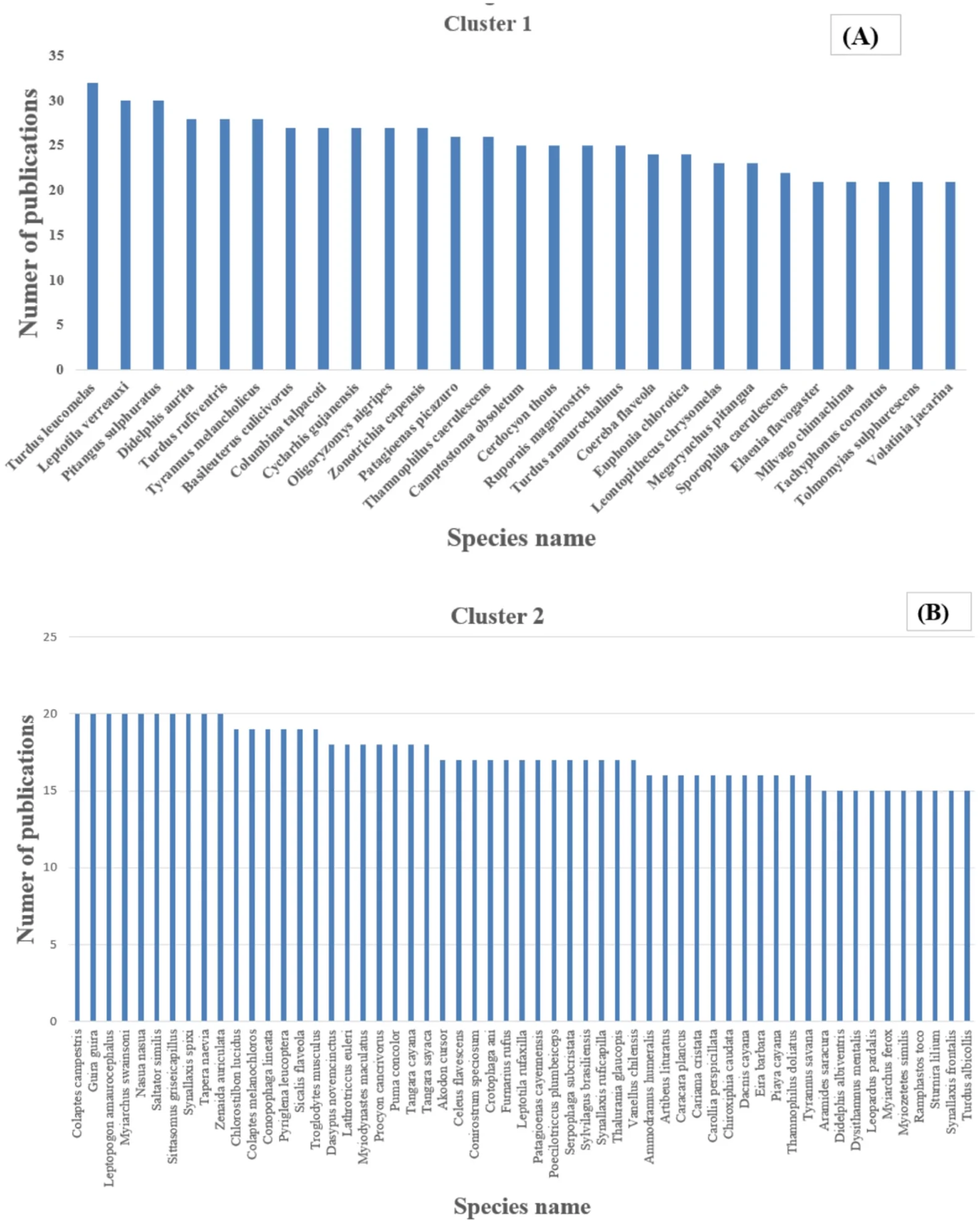

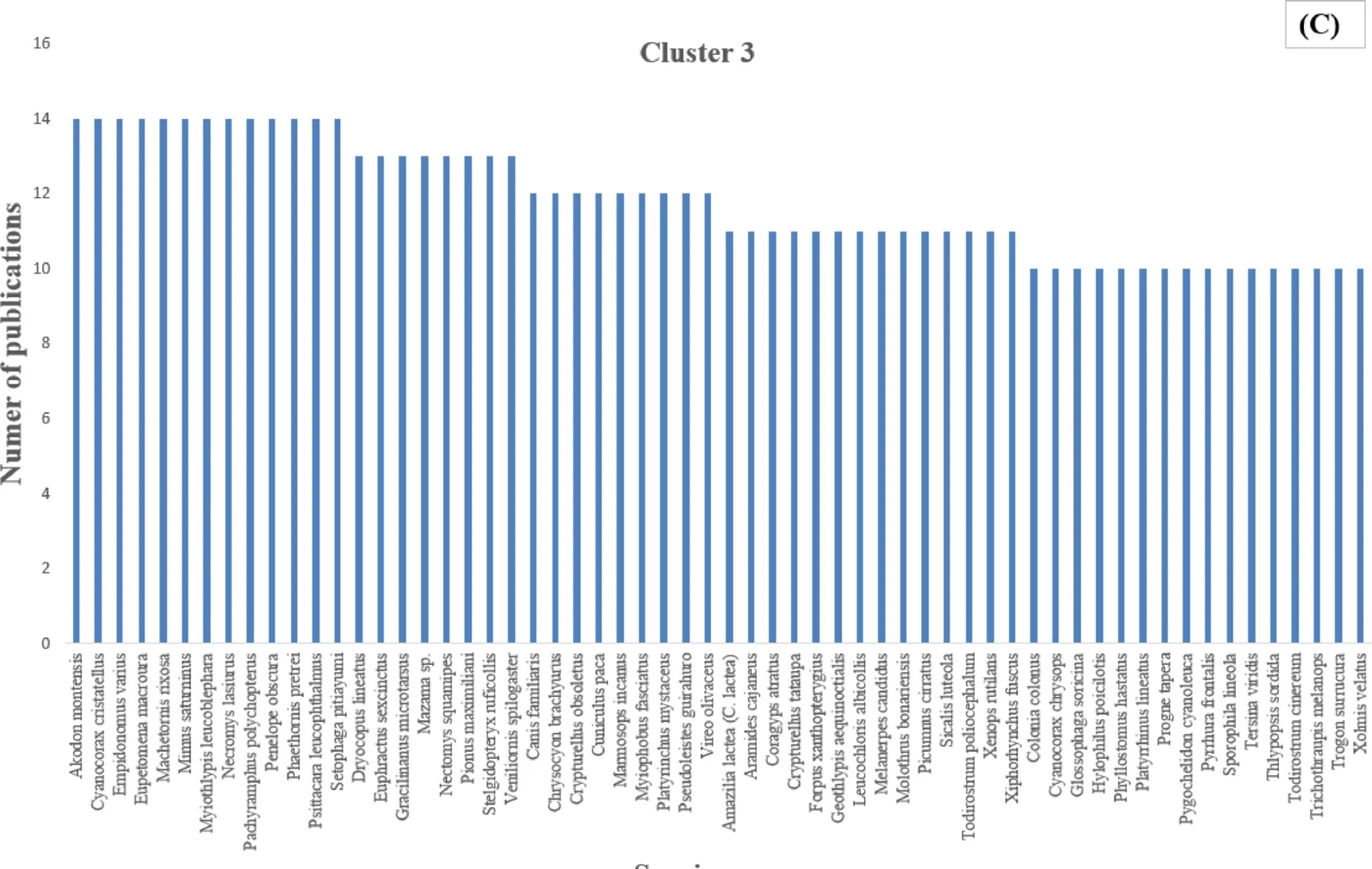
Species-publication relationship clusters in the evidence base. **A** Cluster 1: highly studied species; **B** Cluster 2: moderately studied species. **C** Cluster 3: least studied species

**Fig. 6:**
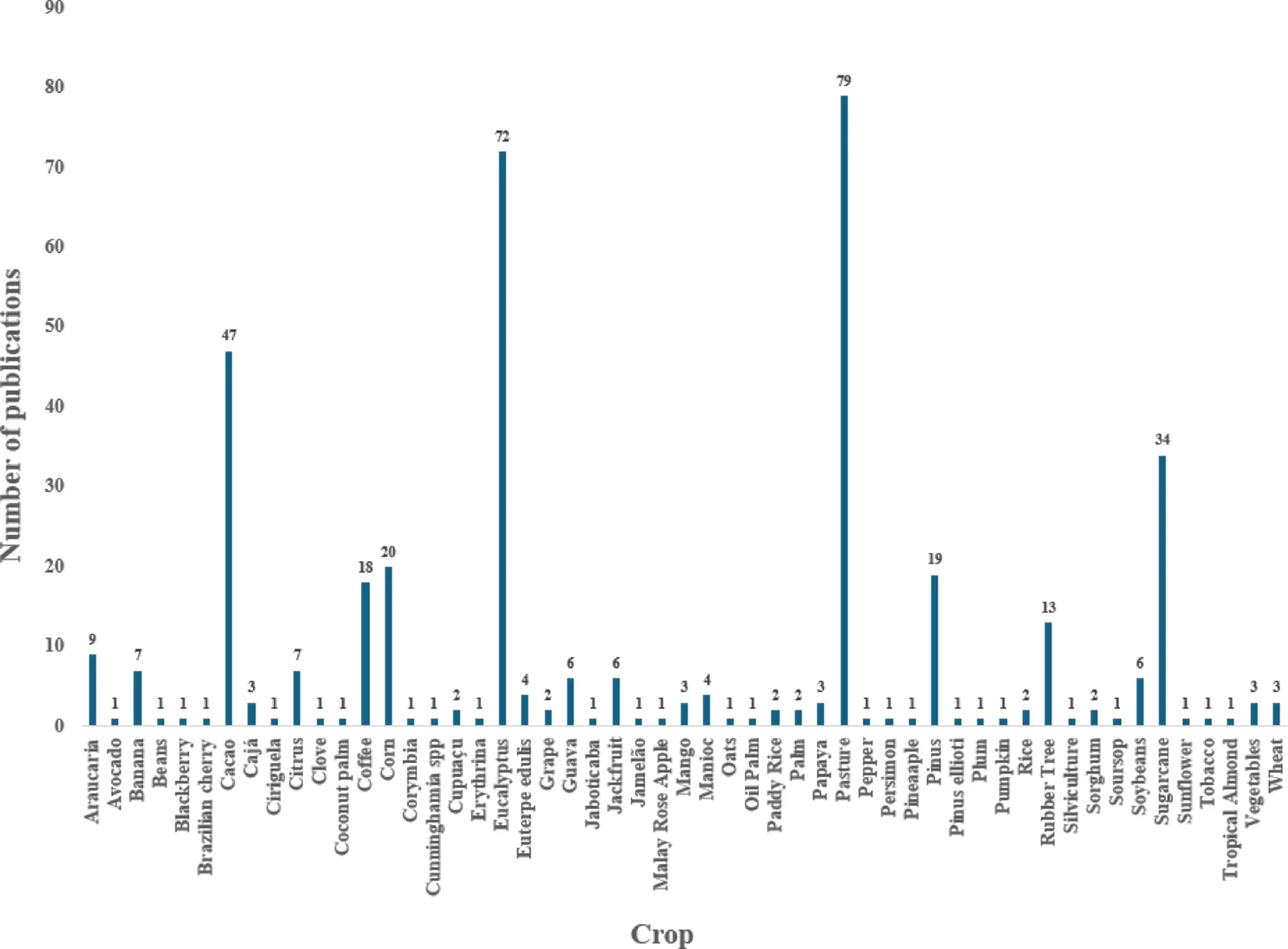
Number of publications for each crop of the evidence base

We have also surveyed the number of publications according to the taxonomic Order and ecosystems. Most of the publications of the evidence base contained observations associated with off-crop areas, therefore for all Orders the association forest-taxonomic Order predominates.

**Fig. 7:**
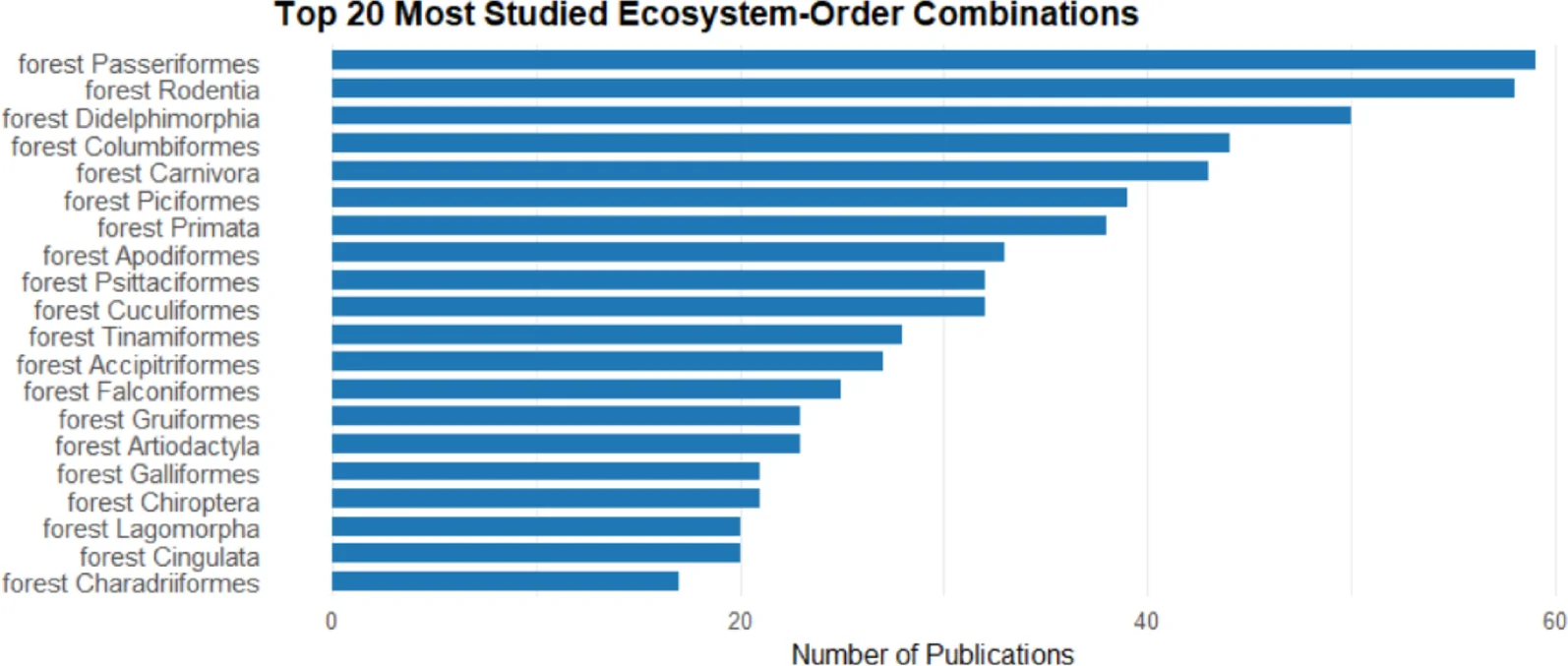
Number of publications according to taxonomic groups and ecosystems

### Mapping the quality of studies relevant to the question

Eligible records were limited to observational and surveyed-based studies. Records that are not primary sources of evidence, such as meta-analysis papers and systematic review papers, were excluded from the systematic map because they could artificially inflate the dataset by duplicating primary sources. Authors typically recorded the presence of species inside crop fields and, in most cases, also in adjacent areas such as riparian zones or forest fragments. While these studies discuss the presence and richness of species, they generally do not describe specific behavioral activities inside the crop fields. These and other knowledge gaps are discussed in further details in the Conclusion section.

#### Conservation status of the species in the evidence base

For each recorded species in the systematic map, we retrieved its conservation status as meta-data according to the IUCN Red List (Fig. 8) [27]. Among the three categories (bats, NFM, birds), the conservation status reported in the evidence base varies. Regarding the most favorable statuses (LC and NT), birds represent the highest proportion in mapped studies, with 97% of species classified as low concern. Additionally, no species in bird meta-data was classified as Data Deficient (DD). Conversely, bat species had the highest percentage of DD status (7%), although most recorded species in the literature have a satisfactory status (89% LC + 3% NT). As for NFM, this taxon displayed the highest records of vulnerable (VU), endangered (EN), and critically endangered (CR) species within the collated evidence, while 77% were classified at low conservation risk (LC + NT).

**Fig. 8:**
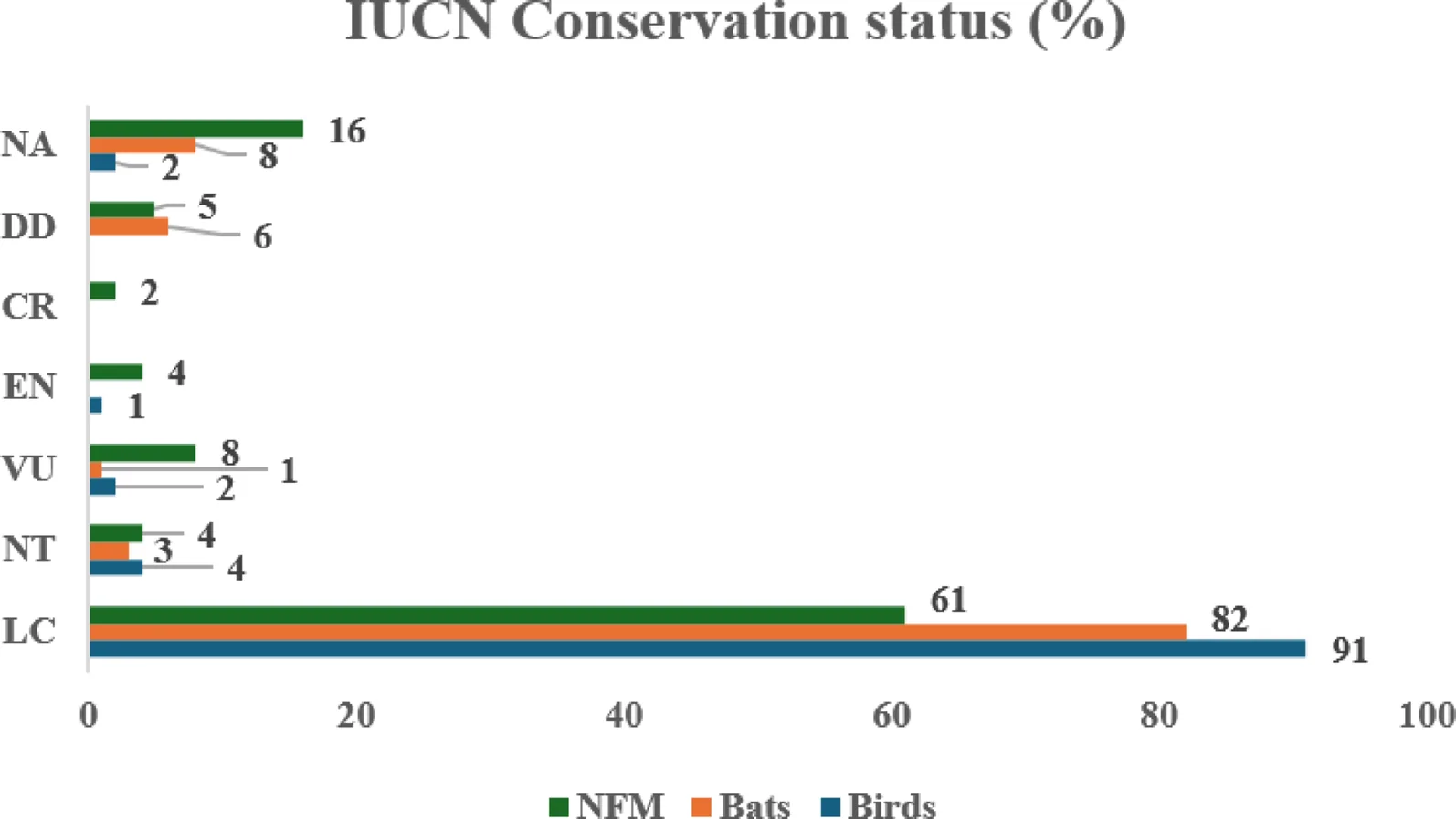
Conservation status of observed species in BAF agricultural landscapes

#### Distribution of research effort across crops and taxonomic groups

To examine how research effort is distributed across different agricultural settings, we displayed the overlap of taxonomic groups recorded per crop type in a Venn diagram (Fig. 9). Overall, non-flying mammals were recorded in 36 different crops, birds in 32, and bats in 20 (*Additional file 6*). The diagram indicates that the most frequently studied crops (those with the highest number of publications, as shown in Fig. 6) are typically associated with records of all three taxonomic groups. Conversely, some crops show a lack of research overlap; for instance, bananas have records only for mammals, while avocados have records only for bats. This highlights a clear knowledge gap in the current evidence base, indicating that further research is needed to determine if other taxa also utilize these specific agricultural environments.

**Fig. 9:**
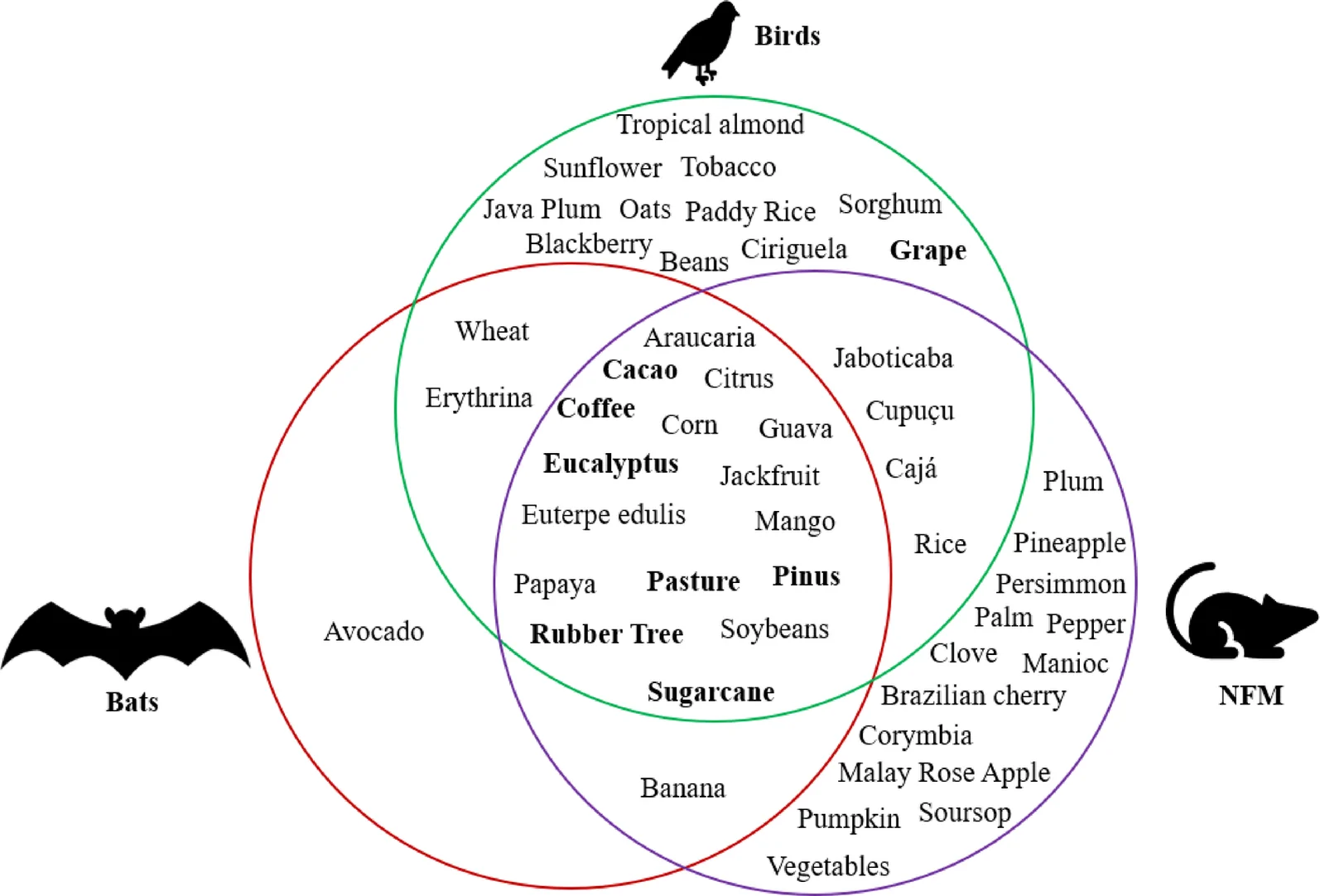
Venn diagram displaying the distribution of records for bats, non-flying mammals (NFM), and birds across crop types as identified in the systematic map. In bold: the ten crops with the highest frequency of records in the evidence base

### Limitations of the systematic map

#### Limitations of the evidence base

The analysis of the evidence base revealed several inherent biases and gaps in the primary literature. Research is heavily concentrated in the Southeast of Brazil, which leaves significant knowledge gaps regarding the agricultural matrix in the Midwest and South regions. Furthermore, the evidence base for specific crops is taxonomically uneven; for instance, while we identified multiple records of bird species in grape plantations, no corresponding records for mammals were found in the surveyed literature. Similarly, no bird records were retrieved for avocados and bananas. These gaps likely reflect a lack of targeted research on these specific crop-taxon combinations rather than a biological absence of species. We also identified specific examples of evidence bias, such as the concentration of bat meta-data in cacao agroforestry systems (cabrucas), while other widely cultivated crops remain underrepresented for this taxon. Additionally, small mammals appear underrepresented in the evidence base, possibly due to the greater sampling difficulty associated with this group compared to large mammals. Finally, most primary records did not focus primarily on the agricultural matrix, as authors often prioritized observations in forest remnants or off-crop areas.

In parallel to this work, the Brazilian environmental agency – Instituto Brasileiro do Meio Ambiente e Recursos Renováveis [IBAMA] – in the effort of discussing environmental risk assessment guidelines for pesticides has released in 2025 a document stating the agency’s concerns regarding the uneven distribution of wildlife studies across Brazilian biomes [32], with a notable concentration in the Atlantic Forest’s South and Southeast regions.

#### Limitations of the review methods

The methodology of this systematic map also presents limitations. First, the search was restricted to records published in English and Portuguese, which may have excluded relevant evidence published in other languages. Second, the search strategy relied on the explicit mention of the “Atlantic Forest” in titles, abstracts, or keywords; consequently, studies conducted within the biome that did not use this specific terminology might not have been retrieved. Furthermore, a significant methodological constraint was the inability to normalize the volume of evidence by sampling effort or the total area of cultivation. Since primary records inconsistently report standardized metrics such as hectares surveyed or trap-hours, our meta-coding was limited to descriptive occurrence counts. Therefore, the findings represent the distribution of available research evidence and research effort rather than a standardized estimation of species’ biological richness across the landscape.

## Conclusions

This systematic map documented the occurrence of 846 species across 48 different crops in the BAF agricultural landscape, with notable records in pastures, eucalyptus plantations, and major commercial crops such as sugarcane and coffee. This compilation of species occurrence data provides a foundation for understanding wildlife presence in these areas, which can inform future research on ecological patterns and the development of conservation and mitigation strategies.

### Implications for research

The outcome of the systematic map documents substantial wildlife presence in agricultural landscapes within the BAF region and it also indicated knowledge gaps as indicated below:

1) Spatial-temporal patterns:

- Limited understanding of the species distribution across different agricultural landscapes.
- Lack of information about the distance between in-crop and off-crop areas.
- There is a need for records correlating wildlife presence with crop development stages (BBCH scale).

2) Behavioral and ecological aspects:

- Insufficient information on foraging and visiting behaviors in agricultural landscapes.
- Limited understanding of whether observed species feed on visited crops.
- Diet composition and proportion obtained from treated areas.
- Food handling behaviors (e.g., fruit and seed peeling).
- Daily food intake patterns.
- Food item assimilation efficiency.
- Preference between treated and untreated crops.

Based on the knowledge clusters and gaps identified in this map, future systematic reviews could focus on:

1. Underrepresented regions/biomes (e.g., caatinga, pampa), crops, and species.
2. The quantitative impact of specific agricultural practices on bird and mammal diversity in high-visitation crops.
3. The role of landscape elements and connectivity specifically for threatened non-flying mammals.
4. Ecological drivers of taxon-specific crop associations, exploring factors like food availability and nesting sites.
5. Requirements for environmental risk assessment (ERA) of pesticides, incorporating evidence of locally documented species to ensure assessments reflect regional ecological realities.
6. Research into the effectiveness and optimal design of biodiversity-friendly practices in high-richness crops (e.g., pastures, eucalyptus, sugarcane, and coffee) to inform and strengthen protection goals and evidence-based management strategies.
7. Knowledge gaps in under-studied crops to strengthen evidence-based biodiversity management.

### Implications for policy/management

This systematic map synthesizes existing evidence on bird and mammal diversity in agricultural landscapes of the BAF. It reveals that research is concentrated on specific crop types (e.g., pasture, eucalyptus) and certain species, with notable geographical imbalances and biases, such as the underrepresentation of small mammals and a focus on off-crop observations. This characterization underscores the need for further evidence syntheses, including quantitative meta-analyses for well-studied areas, and targeted primary research to address identified gaps in crop-taxa associations and geographical coverage. In addition, the meta-data coded from eligible studies provide a regional evidence base that can inform environmental risk assessment frameworks by detailing wildlife presence in agricultural landscapes.

## Supplementary Information

Below is the link to the electronic supplementary material.

Supplementary Material 1.

Supplementary Material 2.

Supplementary Material 3.

Supplementary Material 4.

Supplementary Material 5.

Supplementary Material 6.

Supplementary Material 7.

## Data Availability

The coded datasets generated to produce the Systematic Map are available as additional files. The name of each file is indicated below: Additional file 1 - ROSES form.pdf. Description of data: This file includes the RepOrting standards for Systematic Evidence Synthesis (ROSES) checklist for systematic maps.Additional file 2 - Search strategy and settings.docx. Description of data: This file includes the full search terms, strings for each database, dates of search, and specific search settings used.Additional file 3 - Consistency Check.xlsx. Description of data: This file includes the results of the pilot screening test and the Cohen’s Kappa index calculation.Additional file 4 - Literature Eligibility.xlsx. Description of data: This file includes the list of all exported records, unique identification numbers, and reasons for exclusion at each stage of the screening process.Additional file 5 - Data Coding.xlsx. Description of data: This file includes the codebook and the evidence base with all coded metadata from the eligible publications.Additional file 6 - Supplemental Material. docx. Description of data: This file includes R Studio scripts used for data analysis, Table S1 (Area occupied by BAF), and other supporting tables.Additional file 7 - Publication Cluster.xlsx. Description of data: This file includes the grouping of species into clusters based on the number of individual publications.
