# Supplementary material for: Bird and mammal diversity in agricultural landscapes and adjacent natural vegetation of the Brazilian Atlantic Forest: a systematic map
Source: Environ Evid. 2026 Jul 3;15:11. doi: 10.1186/s13750-026-00390-z (PMC13617712; doi:10.1186/s13750-026-00390-z)
Supplement: Supplementary file 2 [file 13750_2026_390_MOESM2_ESM.docx]

**Additional File 2: Full Search Strategy and Database Settings**

**Content**: This file includes the full search terms, strings for each database, dates of search, and specific search settings used

**Title of the Systematic Map**: Bird and mammal diversity in agricultural landscapes and adjacent natural vegetation of the Brazilian Atlantic Forest: a systematic map

Date of Final Search: February 2025

**1. Search Overview**

To ensure the replicability of this systematic map, this document details the specific settings, filters, and language configurations applied in each database. All searches were conducted without a starting date limit, covering all records up to February 2025.

**2. Database-Specific Settings**

| **Database** | **Language** | **Search Fields** | **Document Type Filters** |
| --- | --- | --- | --- |
| Web of Science (Core Collection) | English | Topic (Title, Abstract, Keywords) | No filters applied |
| Scopus | English | Title, Abstract, Keywords | No filters applied |
| PubMed | English | Title/Abstract (All Fields) | No filters applied |
| BDTD (Biblioteca Digital Brasileira de Teses e Dissertações) | Portuguese | All Metadata Fields | No filters applied |

**3. Search Strings Applied**

***3.1. International Databases (WoS, Scopus, PubMed)***

**Language**: English

**String**: (bird* OR mammal* OR avifaun* OR primate* OR rodent* OR avian OR marsupial* OR faun* OR omnivor* OR carnivor* OR frugivor* OR granivor* OR insectivor* OR bat* OR passerin* OR chiropter*) AND (agricult* OR crop* OR farm* OR pasture OR matrix OR patch* OR corridor* OR “protected area” OR plantation* OR fragment* OR tropic* OR ecosystem* OR agroforest* OR “secondary forest*” OR “forest remnant*” OR “agricultural field*” OR “primary forest*” OR arabl* OR system* OR practice* OR management OR organic OR agroecolog* OR "conservation agriculture" OR "pest management" OR biocontrol OR "urban agriculture" OR till* OR abandonment OR set-aside OR fallow* OR "mixed croplivestock*" OR "integrated crop-livestock*" OR "diversified crop-livestock*" OR "vegetation strip*" OR "insect strip*" OR "flower strip*" OR diversification OR rotation OR "inter-crop" OR covercrop*) AND (“atlantic forest” OR “brazilian atlantic forest” OR riparian OR semi-deciduous OR “brazilian atlantic rainforest*” OR national park* OR tree plantation* OR perenn* OR “perennial crop*” OR neotropic*) AND NOT (caatinga OR cerrado OR amazon OR pampa OR argentina) AND BRAZIL

***3.2. Brazilian Database (BDTD)***

**Language**: Portuguese

**String**: (AVE OR AVIFAUNA OR PASSERIFORME OR MAMÍFERO OR MASTO OR ROEDOR OR PRIMATA OR MARSUPIAL OR FAUNA OR INSETIVOR* OR CARNÍVOR* OR GRANÍVOR* OR FRUGÍVOR* OR ONÍVOR* OR MORCEGO OR QUIRÓPTERO) AND (AGRICULTURA OR AGRO* OR FAZENDA OR CULTIVO OR PLANTAÇÃO OR PAST* OR MATRIZ OR FRAGMENTO OR CORREDOR OR "ÀREA PROTEGIDA" OR TROPICAL OR AGROFLORESTA* OR ECOSISTEMA* OR "FLORESTA SECUNDÁRIA" OR "FLORESTA PRIMÁRIA" OR "FLORESTA REMANESCENTE" OR "CAMPO AGRÍCOLA" OR ARÁVEL OR SISTEMA OR PRÁTICA OR MANEJO OR ORGÂNIC* OR AGROECOLOG* OR "MANEJO DE PRAGAS" OR BIOCONTROLE OR "PLANTIO DIRETO" OR POUSIO OR ROTAÇÃO OR DIVERSIFICAÇÃO OR "COBERTURA VEGETA*" OR "AGRICULTURA URBANA" OR "FAIXA DE FLORES" OR "FAIXA DE INSETOS" OR "FAIXA DE VEGETAÇÃO" OR "CULTURA DE COBERTURA" OR "SISTEMA LAVOURA-PECUÁRIA" OR "INTEGRAÇÃO LAVOURA-PECUÁRIA" OR "LAVOURA-PECUÁRIA DIVERSIFICADA") AND ("MATA ATLÂNTICA" OR "FLORESTA ATLÂNTICA" OR PERENE OR "CULTIVO PERENE" OR NEOTROPICAL OR SEMIDECÍDUA* OR RIPÁRIA NOT CAATINGA NOT CERRAD* NOT AMAZÔNIA NOT PAMPA NOT AMAZON* NOT ARGENTINA)
