# Supplementary material for: Bird and mammal diversity in agricultural landscapes and adjacent natural vegetation of the Brazilian Atlantic Forest: a systematic map
Source: Environ Evid. 2026 Jul 3;15:11. doi: 10.1186/s13750-026-00390-z (PMC13617712; doi:10.1186/s13750-026-00390-z)
Supplement: Supplementary file 3 [file 13750_2026_390_MOESM6_ESM.docx]

**Additional File 6: Supplemental Material**

**Content**: This file includes R Studio scripts used for data analysis, Table S1 (Area occupied by BAF), and other supporting tables

**Title of the Systematic Map:** Bird and mammal diversity in agricultural landscapes and adjacent natural vegetation of the Brazilian Atlantic Forest: a systematic map

Fábio Casallanovo^1,*^, Gustavo Souza Santos^1^, Ana Paola Cione^1^

^1^Syngenta Proteção de Cultivos Ltda

*Corresponding author

email: [fabio.casallanovo@syngenta.com](mailto:fabio.casallanovo@syngenta.com)

**R Studio Scripts**

**Figure 4**

library(readxl)

library(dplyr)

library(ggplot2)

all_pub <- read_excel("C:/Users/u214263/Downloads/Literarature Eligibility.xlsx",

sheet = "All publications")

used_pub <- read_excel("C:/Users/u214263/Downloads/Literarature Eligibility.xlsx",

sheet = "Used publications")

all_pub <- all_pub %>% select(ID = 1, Year = 7)

used_pub <- used_pub %>% select(ID = 1)

filtered_data <- all_pub %>%

semi_join(used_pub, by = "ID")

count_per_year <- filtered_data %>%

group_by(Year) %>%

summarise(Count = n())

ggplot(count_per_year, aes(x = Year, y = Count)) +

geom_col(fill = "steelblue") +

labs(title = "Number of publications used per year",

x = "Publication year",

y = "Number of publications") +

theme_minimal()

ggplot(count_per_year, aes(x = Year, y = Count)) +

geom_col(fill = "steelblue") +

labs(title = "Number of publications used per year",

x = "Publication year",

y = "Number of publications") +

scale_x_continuous(breaks = seq(min(count_per_year$Year),

max(count_per_year$Year),

by = 1)) +	# ticks de 1 em 1 ano

scale_y_continuous(breaks = seq(0, max(count_per_year$Count), by = 1)) + # ticks de 1 em 1

theme_minimal()

install.packages("writexl")

library(writexl)

write_xlsx(count_per_year, "C:/Users/u214263/Downloads/publications_per_year.xlsx")

**Figure 5**

ibrary(readxl)

library(dplyr)

library(ggplot2)

result <- data %>%

group_by(Species) %>%

summarise(num_publications = n_distinct(`Publication ID`)) %>%

mutate(Cluster = case_when(

num_publications > 20 ~ "Cluster 1: >20 publications",

num_publications >= 15 & num_publications <= 20 ~ "Cluster 2: 15–20 publications",

num_publications >= 10 & num_publications <= 14 ~ "Cluster 3: 10–14 publications",

num_publications >= 5 & num_publications <= 9 ~ "Cluster 4: 5–9 publications",

TRUE ~ NA_character_

)) %>%

filter(!is.na(Cluster)) # Optional: remove species with <5 publications

cluster1 <- result %>% filter(Cluster == "Cluster 1: >20 publications")

cluster2 <- result %>% filter(Cluster == "Cluster 2: 15–20 publications")

cluster3 <- result %>% filter(Cluster == "Cluster 3: 10–14 publications")

cluster4 <- result %>% filter(Cluster == "Cluster 4: 5–9 publications")

install.packages("writexl")

library(writexl)

write_xlsx(cluster1, "C:/Users/u214263/Downloads/Cluster1_Publications.xlsx")

write_xlsx(cluster2, "C:/Users/u214263/Downloads/Cluster2_Publications.xlsx")

write_xlsx(cluster3, "C:/Users/u214263/Downloads/Cluster3_Publications.xlsx")

write_xlsx(cluster4, "C:/Users/u214263/Downloads/Cluster4_Publications.xlsx")

File was consolidated as Publcation_Cluster.xlsx, uploaded

**Figure 6**

library(readr)

library(tidyverse)

data <- read_delim("C:/Users/u214263/Downloads/Datacodingstrategy.csv",

delim = ";",

col_names = TRUE)

names(data)

data_long <- data %>%

separate_rows(Crop, sep = ",") %>%

mutate(Crop = str_trim(Crop))

crop_counts <- data_unique %>%

group_by(Crop) %>%

summarise(n_publications = n_distinct(`Publication ID`)) %>%

arrange(desc(n_publications))

print(crop_counts)

print(crop_counts, n = Inf)

library(stringi)

write_csv(crop_counts, "C:/Users/u214263/Downloads/crop_counts_clean.csv")

data <- read_delim("C:/Users/u214263/Downloads/crop_counts_clean.csv",

delim = ";",

col_names = TRUE)

**Figure 7**

library(tidyverse)
library(ggplot2)
library(viridis)
library(treemap)
library(RColorBrewer)

df <- read_delim("C:/Users/s988636/OneDrive - Syngenta/Desktop/BM/systmap.csv", 
                 delim = ";",
                 show_col_types = FALSE)
str(df)
head(df)
names(df)
df_clean <- df %>%
  drop_na() 
str(df_clean)
head(df_clean)
names(df_clean)
heatmap_crop_order <- df_clean %>%
  count(Crop, Order) %>%
  ggplot(aes(x = Crop, y = Order, fill = n)) +
  geom_tile() +
  scale_fill_viridis() +
  theme_minimal() +
  theme(axis.text.x = element_text(angle = 45, hjust = 1)) +
  labs(title = "Distribution of Research Evidence",
       x = "Crop Type",
       y = "Taxonomic Order",
       fill = "Number of Publications")

print(heatmap_crop_order)
heatmap_eco_order <- df_clean %>%
  count(ecosystem, Order) %>%
  ggplot(aes(x = ecosystem, y = Order, fill = n)) +
  geom_tile() +
  scale_fill_viridis() +
  theme_minimal() +
  theme(axis.text.x = element_text(angle = 45, hjust = 1)) +
  labs(title = "Distribution of Research Evidence",
       x = "Ecosystem Type",
       y = "Taxonomic Order",
       fill = "Number of Publications")

print(heatmap_eco_order)
treemap_data <- df_clean %>%
  count(ecosystem, Order)

treemap(
  treemap_data,
  index = c("ecosystem", "Order"),
  vSize = "n",
  type = "index",
  title = "Distribution of Research Evidence",
  palette = rainbow(length(unique(treemap_data$ecosystem)))
)
summary_stats <- df_clean %>%
  group_by(ecosystem, Order) %>%
  summarise(
    n_publications = n_distinct(`Publication ID`),
    .groups = 'drop'
  ) %>%
  arrange(desc(n_publications))
top_20 <- summary_stats %>%
  head(20) %>%
  mutate(
    combination = paste(ecosystem, Order),
    combination = factor(combination, levels = paste(ecosystem, Order))
  ) %>%
  ggplot(aes(x = reorder(combination, n_publications), 
             y = n_publications)) +
  geom_bar(stat = "identity", fill = "#1f77b4", width = 0.7) +
  coord_flip() +
  theme_minimal() +
  theme(
    axis.text.y = element_text(size = 11),
    axis.text.x = element_text(size = 10),
    plot.title = element_text(size = 14, face = "bold"),
    axis.title = element_text(size = 12),
    panel.grid.major.y = element_blank(),
    plot.margin = margin(1, 1, 1, 1, "cm")
  ) +
  labs(
    title = "Top 20 Most Studied Ecosystem-Order Combinations",
    x = "",
    y = "Number of Publications"
  )

 print(top_20)
ggsave("heatmap_crop_order.png", heatmap_crop_order, width = 12, height = 8)
ggsave("heatmap_eco_order.png", heatmap_eco_order, width = 12, height = 8)
ggsave("top_20_combinations.png", top_20, width = 12, height = 8, dpi = 300)
cat("Número de linhas original:", nrow(df), "\n")
cat("Número de linhas após remoção de NA's:", nrow(df_clean), "\n")
cat("Número de linhas removidas:", nrow(df) - nrow(df_clean), "\n")

**Tables**

**Table S1*.*** Area occupied by the Brazilian Atlantic Forest in 2023 per geographic unit

|  | ***BAF Area (ha)*** | ***BAF Area (%)*** |
| --- | --- | --- |
| ***Brazil*** | 16,153,190 | 100 |
| ***Brazilian State/Region*** |  |  |
| Alagoas | 141,836 | 0.88 |
| Bahia | 1,975,434 | 12.23 |
| Ceará | 63,686 | 0.39 |
| Paraíba | 53,755 | 0.33 |
| Pernambuco | 188,574 | 1.17 |
| Piauí | 892,029 | 5.52 |
| Rio Grande do Norte | 12,109 | 0.07 |
| Sergipe | 64,766 | 0.40 |
| ***Total Northeast*** | ***3,392,189*** | ***21.0*** |
| Espirito Santo | 481,719 | 2.98 |
| Minas Gerais | 2,800,145 | 17.33 |
| Rio de Janeiro | 821,087 | 5.08 |
| São Paulo | 2,343,130 | 14.51 |
| ***Total Southeast*** | ***6,446,081*** | ***39.91*** |
| Paraná | 2,312,239 | 14.31 |
| Rio Grande do Sul | 1,090,420 | 6.75 |
| Santa Catarina | 2,179,427 | 13.49 |
| ***Total South*** | ***5,582,086*** | ***34.56*** |
| Goiás | 31,558 | 0.20 |
| Mato Grosso do Sul | 701,275 | 4.34 |
| ***Total Midwest*** | ***732,833*** | ***4.54*** |

**Source:** SOS Mata Atlântica, Atlas da Mata Atlântica 2023-2024. Available at: <https://www.sosma.org.br/sobre/relatorios-e-balancos>

**Table S2**. Non-flying Mammals in BAF Agricultural Landscapes: Characteristics, Conservation Status, and Crop Associations of High-Occurrence Species (≥10 crops)

| **Species name** | **Order** | **Behavior** | **Diet** | **Body weight** | **IUCN** | **Crop association/visitation record** |
| --- | --- | --- | --- | --- | --- | --- |
| *Oligoryzomys nigripes* | Rodentia | Nocturnal | Insectivore/Granivore | 20 g | LC | Araucaria, Banana, Cacao, Clove, Coffee, Corn, Guava, Manioc, Palm tree, Pasture, Pineapple, Pumpkin, Rubber Tree, Sugarcane, Vegetables |
| *Cerdocyon thous* | Carnivora | Nocturnal | Omnivore | 4.5 – 7.7 kg | LC | Araucaria, Banana, Corymbia, Eucalyptus, Jackfruit, Palm Tree, Pastrure, Pinus, Rubber Tree, Sugarcane |
| *Didelphis aurita* | Didelphimorphia | Nocturnal | Omnivore | 1.3-1.5 kg | LC | Banana, Cacao, Cajá, Coffee, Corn, Eucalyptus, Guava, Jackfruit, Manioc, Palm, Pasturep, Pineapple, Pinus, Pumpkin, Rubber Tree, Sugarcane |
| *Cuniculus paca* | Rodentia | Nocturnal | Herbivore | 6-12 kg | LC | Cacao, Cajá, Citrus, Corn, Corymbia, Eucalytus, Jackfruit, Palm Tree, Pasture, Pinus, Soybean, Sugarcane |
| *Procyon cancrivorus* | Carnivora | Nocturnal | Omnivore | 2-12 kg | LC | Cacao, Cajá, Corn, Corymbia, Eucalyptus, Jackfruit, Palm, Pasture, Pinus, Rice, Rubber Tree, Soybean, Sugarcane |
| *Puma yagouaroundi* | Carnivora | Diurnal | Omnivore | 3.5 – 7 kg | LC | Araucaria, Cacao, Cajá, Corn, Corymbia, Eucalyptus, Jackfruit, Palm Tree, Pasture, Pinus, Rubber Tree, Soybeans, Sugarcane |
| *Akodon cursor* | Rodentia | Nocturnal | Omnivore | 40- 45 g | LC | Banana, Cacao, Clover, Eucalyptus, Guava, Manioc, Palm, Pasture, Pineapple, Pumpkin, Rubber Tree, Sugarcane |
| *Dasypus novemcinctus* | Cingulata | Nocturnal | Generalist | 2.5 – 6.5 kg | LC | Banana, Cacao, Citrus, Corn, Corymbia, Eucalyptus, Pasture, Pinus, Rice, Rubber Tree, Soybean, Sugarcane |
| *Eira barbara* | Carnivora | Diurnal | Omnivore | 2.7 – 7.0 kg | LC | Cacao, Cajá, Corn, Corymbia, Eucalyptus, Jackfruit, Palm Tree, Pasture, Pineapple, Rubber Tree, Soybeans, Sugarcane |
| *Euphractus sexcinctus* | Cingulata | Diurnal/Nocturnal | Omnivore | 3.2 – 6.5 kg | LC | Cacao, Cajá, Corn, Corymbia, Eucalyptus, Jackfruit, Palm Tree, Pasture, Pineapple, Rubber Tree, Soybeans, Sugarcane |
| *Hydrochaeris hydrochaeris* | Rodentia | Diurnal | Herbivore | 50 kg | LC | Cacao, Citrus, Corn, Corymbia, Eucalytus, Pasture, Pinus, Rice, Rubber Tree, Soybean, Sugarcane, Vegetables |
| *Leontopithecus chrysomelas* | Primata | Diurnal | Frugivore/Insectivore | 534 – 620 g | EN | Banana, Cacao, Cajá, Cupuaçu, Eucalyptus, Jackfruit, Manioc, Palm Tree, Pasture, Pepper, Rubber Tree, Soursop |
| *Nasua nasua* | Carnivora | Diurnal | Omnivore | 4 kg | LC | Araucaria, Cacao, Citrus, Corn, Corymbia, Eucalyptus, Euterpe edulis, Pasture, Pinus, Rubber Tree, Soybeans, Sugarcane |
| *Pecari tacaju* | Artiodactyla | Diurnal/Nocturnal | Herbivore | 14 – 30 kg | LC | Cacao, Cajá, Corn, Corymbia, Eucalyptus, Jackfruit, Palm Tree, Pasture, Pinus, Rubber Tree, Soybeans, Sugarcane |
| *Sapajus robustus* | Primata | Diurnal | Omnivore | 3.5 kg | EN | Brazilian cherry, Cacao, Cajá, Eucalytus, Euterpe edulis, Jaboticaba, Jackfruit, Malay Rose Apple, Mango, Palm tree, Pasture, Rubber Tree |
| *Sylvilagus brasiliensis* | Lagomorpha | Nocturnal | Herbivore | 934 g | EN | Cacao, Cajá, Citrus, Corn, Corymbia, Eucalyptus, Jackfruit, Pasture, Pinus, Rubber Tree, Soybeans, Sugarcane |
| *Tamandua tetradactyla* | Pilosa | Nocturnal | Insectivore | 4.6 – 5.7 kg | LC | Cacao, Cajá, Corn, Corymbia, Eucalyptus, Jackfruit, Palm Tree, Pasture, Pinus, Rubber Tree, Soybeans, Sugarcane |
| *Leopardus pardalis* | Carnivora | Nocturnal | Generalist | 7 – 15.5 kg | LC | Araucaria, Cacao, Corn, Corymbia, Eucalyptus, Pasture, Pinus, Rice, Rubber Tree, Soybeans, Sugarcane |
| *Myrmecophaga tridactyla* | Pilosa | Diurnal | Insectivore | 18-39 kg | VU | Cacao, Citrus, Corn, Corymbia, Eucalytus, Pasture, Pinus, Rice, Rubber Tree, Soybean, Sugarcane |
| *Cabassous tatouay* | Cingulata | Nocturnal | Insectivore (Myrmecophagous) | 5.35 kg | LC | Cacao, Cajá, Corymbia, Eucalyptus, Jackfruit, Palm Tree, Pasture, Pinus, Rubber Tree, Sugarcane |
| *Mazama americana* | Artiodactyla | Diurnal/Nocturnal | Herbivore | 20 – 55kg | DD | Cacao, Cajá, Corn, Eucalyptus, Jackfruit, Palm Tree, Pasture, Rubber Tree, Soybeans, Sugarcane |
| *Puma concolor* | Carnivora | Nocturnal | Carnivore | 29-120 kg | LC | Araucaria, Cacao, Corn, Corymbia, Eucalyptus, Pasture, Rice, Rubber Tree, Soybeans, Sugarcane |
| *Sus scrofa* | Artiodactyla | Diurnal/Nocturnal | Omnivore | 66-272 kg | LC | Citrus, Coffee, Corn, Corymbia, Eucalyptus, Pasture, Pinus, Rubber Tree, Soybeans, Sugarcane |
| *Tapirus terrestris* | Perissodactyla | Mostly nocturnal | Omnivore | 150-250 kg | VU | Cacao, Citrus, Coffee, Corymbia, Eucalyptus, Papaya, Pasture, Pinus, Rubber Tree, Soybeans, Sugarcane |

**Table S3**. Bats in BAF Agricultural Landscapes: Characteristics, Conservation Status, and Crop Associations of High-Occurrence Species (≥10 crops)

| **Species name** | **Family** | **Subfamily** | **Diet** | **Body weight** | **IUCN** | **Crop association/visitation record** |
| --- | --- | --- | --- | --- | --- | --- |
| *Carollia perspicillata* | Phyllostomidae | Carolliinae | Generalist (fruit, pollen, insects) | 15 g | LC | Avocado, Banana, Cacao, Citrus, Coffee, Corn, Eucalyptus, Erythrina, Euterpe edulis, Guava, Jackfruit, Mango, Papaya, Pasture, Rubber Tree, Soybeans, Sugarcane, Wheat |
| *Sturnira lilium* | Phyllostomidae | Stenodermatinae | Frugivore | 13 – 18 g | LC | Araucaria, Avocado, Banana, Cacao, Citrus, Coffee, Corn, Eucalyptus, Euterpe edulis, Guava, Mango, Papaya, Pasture, Pinus, Rubber Tree, Soybeans, Sugarcane, Wheat |
| *Artibeus lituratus* | Phyllostomidae | Stenodermatinae | Frugivore, Nectarivore | 65.9 g | LC | Avocado, Banana, Cacao, Citrus, Coffee, Corn, Eucalyptus, Euterpe edulis, Guava, Mango, Papaya, Pasture, Soybeans, Sugarcane, Wheat |
| *Phyllostomus hastatus* | Phyllostomidae | Phyllostominae | Omnivore | 80.5-120.5 g | LC | Avocado, Banana, Cacao, Citrus, Coffee, Corn, Eucalyptus, Erythrina, Euterpe edulis, Guava, Jackfruit, Mango, Papaya, Pasture, Rubber Tree, Sugarcane |
| *Platyrrhinus lineatus* | Phyllostomidae | Phyllostominae | Generalist (fruit, pollen, insects) | 22 g | LC | Avocado, Banana, Cacao, Citrus, Coffee, Corn, Eucalyptus, Euterpe edulis, Guava, Mango, Pasture, Papaya, Rubber Tree, Sugarcane |
| *Anoura caudifer* | Phyllostomidae | Glossophaginae | Omnivore | 15.2 g | LC | Avocado, Banana, Cacao, Citrus, Coffee, Corn, Eucalyptus, Euterpe edulis, Guava, Mango, Pasture, Papaya |
| *Artibeus planirostris* | Phyllostomidae | Stenodermatinae | Frugivore | 40 – 60 g | LC | Banana, Cacao, Coffee, Corn, Eucalyptus, Guava, Pasture, Rubber Tree, Soybeans, Sugarcane, Wheat |
| *Eptesicus diminutus* | Vespertilionidae | Vespertilioninae | Insectivores | 4 – 7 g | LC | Avocado, Banana, Citrus, Corn, Eucalyptus, Euterpe edulis, Mango, Papaya, Pasture, Soybeans, Wheat |
| *Glossophaga soricina* | Phyllostomidae | Glossophaginae | Nectarivore, Frugivore | 9.6 g | LC | Avocado, Banana, Cacao, Citrus, Eucalyptus, Euterpe edulis, Guava, Mango, Papaya, Rubber Tree, Sugarcane, |
| *Phyllostomus discolor* | Phyllostomidae | Phyllostominae | Omnivore | 80.5-120.5 g | LC | Avocado, Banana, Cacao, Citrus, Erythrina, Euterpe edulis, Guava, Jackfruit, Mango, Papaya, Rubber Tree, Sugarcane |
| *Myotis albescens* | Vespertilionidae | Vespertilioninae | Insectivore | 4 - 8 g | LC | Avocado, Banana, Cacao, Citrus, Coffee, Eucalyptus, Euterpe edulis, Papaya, Pasture, Rubber Tree |
| *Myotis nigricans* | Vespertilionidae | Vespertilioninae | Insectivore | 4 – 15 g | LC | Avocado, Banana, Cacao, Citrus, Coffee, Eucalyptus, Mango, Papaya, Pasture, Rubber Tree |

**Table S4.** Birds in BAF Agricultural Landscapes: Characteristics, Conservation Status, and Crop Associations of High-Occurrence Species (≥10 crops)

| **Species name** | **Order** | **Diet** | **Body weight** | **IUCN** | **Crop association/visitation record** |
| --- | --- | --- | --- | --- | --- |
| *Zenaida auriculata* | Columbiformes | Granivore | 120-180 g | LC | Araucaria, Citrus, Coffee, Corn, Eucalyptus, Grape, Oats, Pasture, Pinus, Sorghum, Soybeans, Sugarcane, Sunflower, Wheat |
| *Columbina talpacoti* | Columbiformes | Granivore | 35-56 g | LC | Cacao, Citrus, Coffee, Corn, Eucalyptus, Grape, Guava, Paddy Rice, Pasture, Rubber Tree, Soybeans, Sugarcane, Wheat |
| *Euphonia chlorotica* | Passeriformes | Frugivore | 8 g | LC | Araucaria, Cacao, Citrus, Coffee, Corn, Eucalyptus, Grape, Pasture, Pinus, Rubber Tree, Soybeans, Sugarcane, Wheat |
| *Leptotila rufaxilla* | Columbiformes | Generalist | 115-183 g | LC | Araucaria, Cacao, Citrus, Coffee, Corn, Eucalyptus, Grape, Pasture, Pinus, Rubber Tree, Soybeans, Sugarcane, Wheat |
| *Leptotila verreauxi* | Columbiformes | Granivore/Frugivore | 160-215 g | LC | Araucaria, Cacao, Citrus, Coffee, Corn, Eucalyptus, Grape, Pasture, Pinus, Rubber Tree, Soybeans, Sugarcane, Wheat |
| *Pitangus sulphuratus* | Passeriformes | Omnivore | 52-68 g | LC | Araucaria, Cacao, Citrus, Coffee, Corn, Eucalyptus, Grape, Pasture, Pinus, Rubber Tree, Soybeans, Sugarcane, Wheat |
| *Ramphastos toco* | Piciformes | Omnivore | 540 g | LC | Blackberry, Citrus, Coffee, Corn, Eucalyptus, Grape, Guava, Java Plum, Mango, Pasture. Soybeans, Sugarcane, Wheat |
| *Turdus leucomelas* | Passeriformes | Insectivore | 69 g | LC | Araucaria, Cacao, Citrus, Coffee, Corn, Eucalyptus, Grape, Pasture, Pinus, Rubber Tree, Soybeans, Sugarcane, Wheat |
| *Turdus rufiventris* | Passeriformes | Frugivore/Insectivore | 68-80 g | LC | Araucaria, Cacao, Citrus, Coffee, Corn, Eucalyptus, Grape, Pasture, Pinus, Rubber Tree, Soybeans, Sugarcane, Wheat |
| *Celeus flavescens* | Piciformes | Frugivore/Insectivore | 110-165 g | LC | Cacao, Citrus, Coffee, Corn, Eucalyptus, Grape, Pasture, Pinus, Rubber Tree, Soybeans, Sugarcane, Wheat |
| *Milvago chimachima* | Falconiformes | Omnivore | 277-335 g | LC | Araucaria, Cacao, Citrus, Coffee, Corn, Eucalyptus, Grape, Pasture, Pinus, Soybeans, Sugarcane, Wheat |
| *Patagioenas picazuro* | Columbiformes | Granivore/Frugivore | NA | LC | Araucaria, Citrus, Coffee, Corn, Eucalyptus, Grape, Paddy Rice, Pasture, Pinus, Soybeans, Sugarcane, Wheat |
| *Rupornis magnirostris* | Accipitriformes | Generalist | 206-350 g | LC | Araucaria, Cacao, Citrus, Coffee, Corn, Eucalyptus, Grape, Pasture, Rubber Tree, Soybeans, Sugarcane, Wheat |
| *Tangara cayana* | Passeriformes | Frugivore/Insectivore | 20 g | LC | Cacao, Citrus, Corn, Eucalyptus, Grape, Guava, Pasture, Pinus, Rubber Tree, Soybeans, Sugarcane, Wheat |
| *Turdus amaurochalinus* | Passeriformes | Frugivore/Insectivore | 57.4-59.5 | LC | Cacao, Citrus, Coffee, Corn, Eucalyptus, Grape, Pasture, Pinus, Rubber Tree, Soybeans, Sugarcane, Wheat |
| *Zonotrichia capensis* | Passeriformes | Generalist | 20 -25 g | LC | Araucaria, Citrus, Coffee, Corn, Eucalyptus, Grape, Paddy Rice, Pasture, Pinus, Soybeans, Sugarcane, Wheat |
| *Basileuterus culicivorus* | Passeriformes | Insectivore | 10.5 g | LC | Araucaria, Citrus, Coffee, Corn, Eucalyptus, Grape, Pasture, Pinus, Soybeans, Sugarcane, Wheat |
| *Cariama cristata* | Cariamiformes | Carnivore | 1.4 kg | LC | Citrus, Coffee, Corn, Eucalyptus, Grape, Pasture, Pinus, Soybeans, Sugarcane, Vegetables, Wheat |
| *Chiroxiphia caudata* | Passeriformes | Frugivore | 25.6 g | LC | Araucaria, Citrus, Coffee, Corn, Eucalyptus, Grape, Pasture, Pinus, Soybeans, Sugarcane, Wheat |
| *Coereba flaveola* | Passeriformes | Generalist | 8 – 10 g | LC | Cacao, Coffee, Corn, Erythrina, Eucalyptus, Grape, Jackfruit, Pasture, Pinus, Rubber Tree, Sugarcane |
| *Cyclarhis gujanensis* | Passeriformes | Insectivore | 28 g | LC | Araucaria, Citrus, Coffee, Corn, Eucalyptus, Grape, Pasture, Pinus, Soybeans, Sugarcane, Wheat |
| *Piaya cayana* | Cuculiformes | Insectivore | 95-120 g | LC | Araucaria, Citrus, Coffee, Corn, Eucalyptus, Grape, Pasture, Rubber Tree, Soybeans, Sugarcane, Wheat |
| *Pyriglena leucoptera* | Passeriformes | Insectivore | 25- 34 g | LC | Araucaria, Cacao, Coffee, Corn, Eucalyptus, Erythrina, Grape, Jackfruit, Pasture, Rubber Tree, Sugarcane |
| *Synallaxis spixi* | Passeriformes | Insectivore | 14 g | LC | Araucaria, Citrus, Coffee, Corn, Eucalyptus, Grape, Pasture, Pinus, Soybeans, Sugarcane, Wheat |
| *Tachyphonus coronatus* | Passeriformes | Generalist | 26-33 | LC | Araucaria, Citrus, Coffee, Corn, Eucalyptus, Grape, Pasture, Pinus, Soybeans, Sugarcane, Wheat |
| *Volatinia jacarina* | Passeriformes | Granivore/Insectivore | 11 g | LC | Araucaria, Citrus, Coffee, Corn, Eucalyptus, Grape, Paddy rice, Pasture, Soybeans, Sugarcane, Wheat |
| *Amazilia lactea* | Apodiformes | Nectivore | 3 – 6 g | LC | Citrus, Coffee, Corn, Eucalyptus, Grape, Pasture, Pinus, Soybeans, Sugarcane, Wheat |
| *Caracara plancus* | Falconiformes | Generalist | 834 g | LC | Cacao, Citrus, Coffee, Corn, Eucalyptus, Grape, Pasture, Soybeans, Sugarcane, Wheat |
| *Colaptes campestris* | Piciformes | Insectivore | 150 g | LC | Araucaria, Citrus, Coffee, Corn, Eucalyptus, Grape, Pasture, Soybeans, Sugarcane, Wheat |
| *Cyanocorax chrysops* | Passeriformes | Insectivore/Frugivore | NA | LC | Araucaria, Cajá, Corn, Eucalyptus, Guava, Jaboticaba, Papaya, Pasture, Pinus, Sugarcane |
| *Furnarius rufus* | Passeriformes | Insectivore | 49 g | LC | Citrus, Coffee, Corn, Eucalyptus, Grape, Pasture, Pinus, Soybeans, Sugarcane, Wheat |
| *Guira guira* | Cuculiformes | Carnivore | 113- 168.3 g | LC | Citrus, Coffee, Corn, Eucalyptus, Grape, Pasture, Pinus, Soybeans, Sugarcane, Wheat |
| *Leucochloris albicollis* | Apodiformes | Nectivore | 5 – 8 g | LC | Araucaria, Citrus, Coffee, Corn, Eucalyptus, Grape, Pasture, Pinus, Soybeans, Wheat |
| *Melanerpes candidus* | Piciformes | Generalist | 98 – 136 g | LC | Araucaria, Citrus, Coffee, Corn, Eucalyptus, Grape, Pasture, Soybeans, Sugarcane, Wheat |
| *Penelope obscura* | Galliformes | Frugivore | 1.0 – 1.2 kg | LC | Araucaria, Beans, Coffee, Corn, Eucalyptus, Grape, Guava, Pasture, Sugarcane, Vegetables |
| *Setophaga pitiayumi* | Passeriformes | Generalist | 7.5 g | LC | Araucaria, Citrus, Coffee, Corn, Eucalyptus, Grape, Pasture, Pinus, Soybeans, Wheat |
| *Sporophila caerulescens* | Passeriformes | Granivore | 10.5 g | LC | Araucaria, Citrus, Coffee, Corn, Eucalyptus, Grape, Pasture, Soybeans, Sugarcane, Wheat |
| *Theristicus caudatus* | Pelecaniformes | Generalist | 1.46 – 1.55 kg | LC | Araucaria, Citrus, Coffee, Corn, Grape, Pasture, Pinus, Soybeans, Sugarcane, Wheat |
| *Troglodytes musculus* | Passeriformes | Insectivore | 10 -12 g | LC | Citrus, Coffee, Corn, Eucalyptus, Grape, Pasture, Pinus, Soybeans, Sugarcane, Wheat |
